# Cocirculation of Dengue Serotypes, Delhi, India, 2003

**DOI:** 10.3201/eid1202.050767

**Published:** 2006-02

**Authors:** Lalit Dar, Ekta Gupta, Priyanka Narang, Shobha Broor

**Affiliations:** * All India Institute of Medical Sciences, New Delhi, India

**Keywords:** Dengue virus, epidemiology, outbreaks, India, letter

**To the Editor:** Delhi, in the northern part of India, has had outbreaks of dengue caused by various dengue virus types in 1967, 1970, 1982, 1988, and 1996 (*1**–**5*). In 1988, for the first time, a few cases of dengue hemorrhagic fever (DHF) were seen (*4*). Subsequently, we reported the largest outbreak of DHF/dengue shock syndrome (DSS) in Delhi in 1996 and confirmed dengue virus type 2 as the etiologic agent (*5*).

We report the results of virologic testing of samples received at the All India Institute of Medical Sciences from patients with suspected dengue fever or denguelike illness from Delhi and its adjoining areas during a 2003 outbreak of dengue. According to the World Health Organization (*6*), 2,185 laboratory-confirmed cases were reported during this outbreak.

Of the blood samples received by the virology laboratory, 42 were received on ice from patients with acute denguelike illness. Serum was separated aseptically and stored at –70°C. The standard method of virus cultivation, which used the C6/36 clone of the *Aedes albopictus* cell line, was followed with some modifications (*7*). On days 5 and 10, harvested cells were tested by an indirect immunofluorescence assay (IFA) using monoclonal antibodies to dengue virus types 1–4 (provided by the Centers for Disease Control and Prevention, Atlanta, Georgia, USA, during the 1996 outbreak). If IFA results were negative for dengue viruses on first passage, a second passage was made, and cells were again harvested on days 5 and 10 for IFA. The 4 dengue virus types (obtained from the National Institute of Virology, Pune, India) were included as positive controls, and uninfected C6/36 cells were kept as negative controls.

Dengue virus could be isolated in C6/36 cells from 8 (19%) of 42 samples processed for virus isolation (Table). Of the 8 isolates, two each were identified as dengue virus types 1 and 2, three as type 3, and one as type 4. All but one isolate were from patients with uncomplicated dengue fever. One dengue type 2 isolate was obtained from a 7-year-old boy with secondary dengue infection and DHF/DSS. The ages of culture-positive patients ranged from 5 to 62 years, with a median of 22 years. These patients were equally distributed between children (<12 years) and adults. The male-to-female ratio for these 8 patients was 5:3. The duration of fever at the time of viral isolation was 1–5 days, with a median of 3 days.

**Table:** Culture-positive dengue patients*

Age (y)/sex	Dengue type isolated	Secondary infection (anti–dengue IgG antibodies + by ELISA)	Duration of fever (d)
9/M	DENV-1	Yes	4
25/F	DENV-3	No	4
7/M	DENV-2	Yes	5
7/F	DENV-4	No	1
40/F	DENV-1	Yes	3
62/M	DENV-2	Yes	3
39/M	DENV-3	Yes	2
5/M	DENV-3	No	3

*ELISA, enzyme-linked immunosorbent assay; IgG, immunoglobulin G.

All previous outbreaks in Delhi have occurred during the monsoon (rainy) season between August and November and subsided with the onset of winter. We recently reported the results of serologic testing during the 2003 outbreak, which also occurred from September to November, with a peak in mid-October 2003 (*8*). This outbreak was milder than the 1996 outbreak, with less illness and death; most patients had uncomplicated dengue fever, and only a few had DHF/DSS. Of the 874 serum samples that we tested, 456 (52.3%) were positive for dengue-specific immunoglobulin M antibodies by enzyme-linked immunosorbent assay (Panbio, Sinnamon Park, Queensland, Australia), and more than one third of these were from patients in the 21- to 30-year age group (*8*).

Dengue virus types 1, 2, and 3 have all been isolated during previous dengue outbreaks in Delhi, but a particular type has always predominated. During the 1996 outbreak of DHF/DSS, we had 26 isolates of dengue virus type 2, but only 1 isolate was identified as dengue type 1 (*5*). However, we subsequently showed that dengue virus type 1 continued to circulate during the postepidemic period and became the predominant strain (*9*). Dengue virus type 3 has recently reemerged in South Asia, including north India (*10*). We now report this culture-confirmed outbreak of dengue from Delhi, during which the simultaneous transmission of all 4 dengue virus types has been demonstrated for the first time in India, with no particular type predominating. This finding suggests that dengue is now truly endemic in this region.
